# Origins of Self-Fertility in Three *Caenorhabditis* Nematodes

**DOI:** 10.3390/biology15110876

**Published:** 2026-06-02

**Authors:** Montana Bobinski, James Kennedy, David Pilgrim, Ronald E. Ellis

**Affiliations:** 1 Department of Biological Sciences, University of Alberta, Edmonton, AB T6G 2E9, Canada; 2 Department of Cell & Molecular Biology, Rowan-Virtua School of Osteopathic Medicine, Stratford, NJ 08084, USA

**Keywords:** evolution, nematode, hermaphrodite, *Caenorhabditis*, complex trait

## Abstract

How does novelty arise during evolution? Over the past few decades, work from many labs has developed a detailed genetic and cellular picture of how nematode mating systems were altered to yield self-fertile hermaphrodites from ancestral females. Since most analyses involve *Caenorhabditis*, this review focuses on three androdiecious species from this genus. In each, male spermatogenesis and sperm activation programs were co-opted for use in *XX* animals. Since each species followed a unique pathway to self-fertility, comparative analyses reveal how these regulatory pathways changed to produce new traits. Moreover, ecological studies are beginning to provide models for how selection and population structure might have interacted with these regulatory changes through a stepwise process. Taken together, our new understanding of how this complex trait originated combines evolutionary, genetic and developmental principles into a robust picture of evolutionary change. Comparing these results from *Caenorhabditis* with studies of more distant groups of nematodes provides further support for many of the conclusions.

## Simple Summary

Although most nematodes have male/female mating systems, self-fertile hermaphrodites have evolved independently on numerous occasions. This review summarizes how three *Caenorhabditis* species produce hermaphrodites, focusing on changes to the sex-determination process in germ cells, and on sperm activation. These changes allowed *XX* animals in each species to co-opt male regulatory programs so that they could produce their own sperm and use them to self-fertilize their own oocytes. Finally, these results are considered in the light of population structures and ecology, and compared with information from other groups of nematodes, to prepare a model for the evolutionary process that led to self-fertility.

## 1. Evolution, Developmental Biology and the Origin of Complex Traits

Darwin’s Theory of Evolution is based on a few key observations [1]. He noted that individuals produce many more offspring than survive to adulthood, and that these offspring differ in heritable traits. Hence, he inferred that individuals with favorable traits should be more likely to survive and reproduce, and that these favorable traits should become more common over time. Decades later, the modern synthesis fused the new field of genetics with that of evolution, leading to sophisticated models of how allele frequencies change in populations, and how these changes alter traits (reviewed by [2,3]).

Unfortunately, the modern synthesis could not predict which molecular changes would produce differences in traits large enough to influence survival or reproduction. Addressing this problem required incorporating developmental biology into the theory to elucidate how genes control phenotypes (reviewed by [4]). This approach offered further rewards, like the opportunity to study how regulatory pathways themselves change over time (e.g., [5]), or how robustness allows the accumulation of hidden genetic variation [6]. An additional benefit is that incorporating developmental biology helps us address one of the most profound questions Darwin confronted—how complex traits originate.

Complex traits are produced by the coordinated action of many genes shaping the functions of multiple cells to produce a trait or structure. Familiar examples include eyes (reviewed by [7,8,9]), wings (for a review of insect wings see [10], for pterosaurs see [11]) and bird feathers (reviewed by [12]). Although evolutionary theory easily explains how simple traits change incrementally, such as fur becoming lighter in a new environment [13], describing how multiple factors come together to make a wing is more difficult.

As a result, many researchers have been studying recent morphological innovations, which are easier to dissect genetically. Much of this work is done with insects, because of their diversity and genetic tractability. For example, the formation and evolution of butterfly wing spots can elucidate how conserved signal transduction pathways are recruited to produce and shape new traits (e.g., [14,15]). Similarly, beetle horns are rapidly evolving structures that are sex- and species-specific, and often develop in response to cues like nutritional status (reviewed by [16,17,18]). And treehoppers provide a model for probing the origins of a novel structure often used for camouflage [19].

When considering how complex traits like these arose, Darwin proposed that each originally served a separate purpose that was co-opted to carry out a new function [1]. Although definitions vary, we use ‘co-option’ to refer to traits that are repurposed for use in a new context. For example, swim bladders evolved for flotation in fish, but have often been repurposed for producing sound [20]. Furthermore, wing-like appendages in treehoppers have been co-opted to form elaborate types of camouflage [19]. Finally, analyses of beetle horns and butterfly wing spots reveal co-option of existing signal transduction pathways to initiate growth or color patterns in new locations [21]. This process of co-option is also seen at the level of individual genes [22].

Although co-option is clearly involved in producing novel traits, many questions remain about how it works in practice. To what extent does co-option rely on duplication and divergence, as is often seen in gene evolution or with insect appendages? Can co-option recruit more than one trait at a time, and if so, how are changes in different systems coordinated? Finally, how does interaction between changes in external selective forces and in intrinsic genetic mutations influence co-option?

Our review synthesizes work on *Caenorhabditis* nematodes to explain how molecular and developmental changes in gene regulation produce novel traits and then shape them during evolution. Besides co-option, we also consider developmental constraints, which depend on the genetic and regulatory architecture of body plans and signaling pathways [23,24]. These constraints sometimes prevent and at other times facilitate specific changes.

## 2. *Caenorhabditis* Mating Systems Provide a Model for the Evolution of Novel Traits

Nematodes of the genus *Caenorhabditis* provide a valuable alternative to insect models for studying evolutionary questions. Like insects, many nematode species are easy to culture and study in the laboratory (e.g., [25]). Of particular importance, numerous morphological traits differ in interesting ways between species, including vulval development, which is controlled by interacting signal transduction pathways; male tail development, which depends on a conserved transcription factor program; and early embryonic development (reviewed by [26]). Moreover, nematodes have proven fruitful for examining evolutionary phenomena like developmental constraints [27,28].

**Figure 1:**
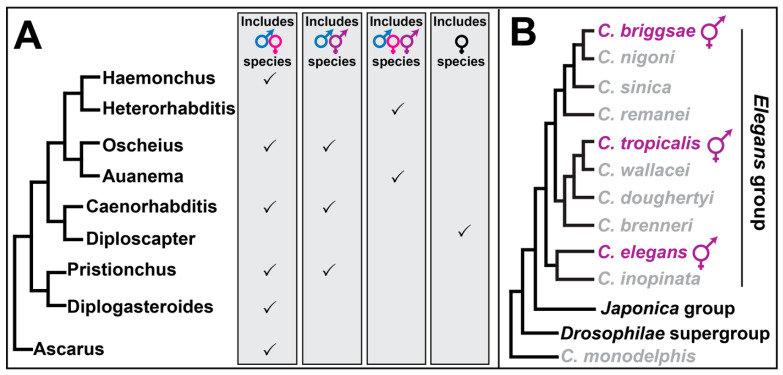
Frequent alteration of nematode mating systems during evolution. (**A**) Partial phylogeny of the Rhabditidae, showing relevant genera. It is largely based on work by Haag et al. [26], but with modifications incorporating data by Tandonnet et al. [29] and Qing et al. [30]. The ancestral state was male/female. From left to right, the column headings indicate the following: male/female (diecious), male/hermaphrodite (androdiecious), male/female/hermaphrodite, and parthenogenetic. (**B**) Phylogeny of the Elegans group of *Caenorhabditis*, based on work by Kiontke et al. [33]. Species in gray are male/female, and those in purple are male/hermaphrodite.

Crucially, nematodes also provide an excellent model for learning how reproductive traits evolve. This topic is of great importance not only for individual animals, but for entire population structures. Species in the phylum Nematoda have undergone frequent changes in mating systems, often from male/female to male/hermaphrodite reproduction, but sometimes to other systems, including parthenogenesis (Figure 1A, reviewed by [26], but see also [29,30]). Just within the genus *Caenorhabditis*, self-fertile hermaphrodites have evolved on three separate occasions (Figure 1B, [31,32,33]). Furthermore, extensive genomic resources are available for this group, from the complete sequences for *C. elegans* [34] and *C. briggsae* [35,36] to assemblies for many new species (e.g., [37]).

## 3. What Is Required to Produce a *Caenorhabditis* Hermaphrodite?

**Figure 2:**
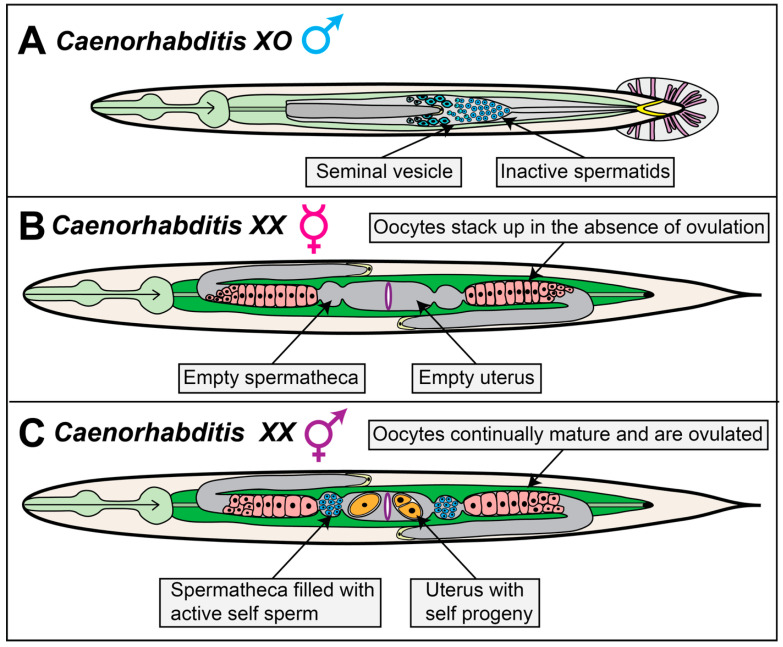
Diagrams of reproductive structures in each sex of *Caenorhabditis*. Anterior is left and ventral is facing up. (**A**) Males only make spermatids, which remain inactive in the seminal vesical until mating (small blue cells). They activate as they pass through the Vas deferens during mating. (**B**) Virgin females only make oocytes (pink), which remain in the ovary until mating. (**C**) Hermaphrodites resemble females but produce sperm late in larval development, which they activate and store for self-fertilization (blue). The vulva is purple, and embryos are orange.

*C. elegans*, *C. briggsae* and *C. tropialis* are androdiecious—they produce males and hermaphrodites, which either self-fertilize or mate with males. In essence, these hermaphrodites are females that (1) make their own spermatids, (2) activate these spermatids into motile sperm that fertilize oocytes, and (3) store these sperm until needed (Figure 2). Studies of the male/female species *C. remanei* [38] and *C. nigoni* [39] showed that causing *XX* animals to make spermatids prior to beginning oogenesis and providing a sperm activation signal were necessary and sufficient to make them self-fertile, like hermaphrodites. No manipulations were needed to facilitate the storage of sperm, since the females have spermathecae between the ovary and the uterus for storing male sperm after copulation ([40], reviewed by [41]). Thuse, these spermathecae might favor the evolution of self-fertility.

## 4. *C. elegans* Somatic Sex Determination

The most well-known nematode is the male/hermaphrodite species *C. elegans* [42]. It is one of the leading genetic models for development (reviewed by [25]) and specifically for studying sex determination (reviewed by [43,44]). This provides an enormous advantage for studying related *Caenorhabditis* species and their changing mating systems. First, the wealth of information known about *C. elegans* sex determination and sperm activation provides an entry point for analyzing mating systems in related nematodes (below, reviewed by [45]). Second, many tools developed for genetic analysis in *C. elegans* can be adapted to its relatives (e.g., mutational analysis, [46], RNAi, [47], deletion screening, [48], gene editing, [49]). Third, a detailed comparison of sex determination in the *C. elegans* soma and germ line suggests models for how hermaphrodite development might have evolved.

In *C. elegans*, *XO* animals become males and *XX* animals become hermaphrodites. Sex determination consists of three steps—interpreting the *X:Autosome* ratio to initiate proper sexual development and dosage compensation, coordinating sexual development in cells throughout the body by an extracellular signal, and interpreting that signal within each cell to control the master transcription factor TRA-1, which promotes female development. Although sex determination in the soma and in germ cells are related, they are not identical and will be treated separately here.

### 4.1. XOL-1 Interprets the X:Autosome Ratio and Controls the SDC Complex

For both soma and germ line, the initial sex-determination signal depends on the ratio of *X* chromosomes to autosomes [50]. This ratio regulates the activity of *xol-1*, which is normally expressed in *XO* animals to promote male development and repressed in *XX* animals to allow hermaphrodite development [51]. This regulation depends on competition between autosomal factors that promote *xol-1* activity and *X*-linked ones that repress it (reviewed by [52]). When XOL-1 is expressed, it blocks the action of the SDC complex, which would otherwise promote the expression of dosage compensation genes and repress the expression of *her-1*. Since *xol-1* control of sex determination has been identified in both *C. elegans* and *C. briggsae*, it is likely that the control of *xol-1* expression via an *X*-chromosome counting system is a conserved trait across *Caenorhabditis* [53,54,55].

### 4.2. The HER-1 Signal Inhibits the Activity of the TRA-2 Receptor

The *her-1* gene is required for *XO* animals to become male [56]. It encodes a small protein that acts as a sex hormone [57]. HER-1 is not secreted from a single source, but is instead produced by many cells, so that sex is determined by a broad consensus of tissues [58]. To promote male fates, HER-1 binds to and inhibits the TRA-2 receptor [59,60]. This large transmembrane protein [61] is required for hermaphrodite development [62]. If HER-1 is not present, TRA-2’s C-terminal intracellular domain is cleaved off by the calpain protease TRA-3 [63,64]. Once released, this domain, known as TRA-2_ic_, is free to bind FEM-3 [65] or TRA-1 [66,67]. In the soma, the critical interaction is with FEM-3 (below).

### 4.3. TRA-2 Binds FEM-3 to Regulate the FEM E3 Ubiquitin Ligase Complex

Within each cell, the *fem-1*, *fem-2* and *fem-3* genes promote male development [68,69,70,71]. FEM-1 is a conserved ankyrin-repeat protein [72], FEM-2 is a protein phosphatase type 2C [73] and FEM-3 represents a novel class of protein [74]. These proteins act with CUL-2 as part of a ubiquitin ligase complex that targets the transcription factor TRA-1 for degradation [75]. Because all three proteins are needed for the complex to work, the ability of TRA-2_ic_ to bind FEM-3 can lower the activity of the FEM complex enough that TRA-1 is free to promote hermaphrodite fates [66].

### 4.4. The Gli Transcription Factor TRA-1 Is Cleaved to Form a Repressor of Male Genes

The master transcription factor for nematode sex determination is TRA-1, the sole Gli protein in these animals [76]. It is required for hermaphrodite development [62], and gain-of-function mutations (gf) cause all animals, including the *XO*, to become true females [56,77]. These gf mutations cluster in a small regulatory site N-terminal to the zinc fingers [78], which might be involved in regulation by the FEM complex. TRA-1 normally represses genes needed for male development like *egl-1* [79], *mab-3* [80] and numerous others [81]. This repression is mediated by a truncated form often known as TRA-1^100^, which lacks the C-terminus [82]. Thus, we will refer to it as TRA-1^rep^. Visualization of GFP::TRA-1 in the developing nervous system shows that its expression is determined on a cell-by-cell basis, as needed for sexual differentiation [83].

**Figure 3:**
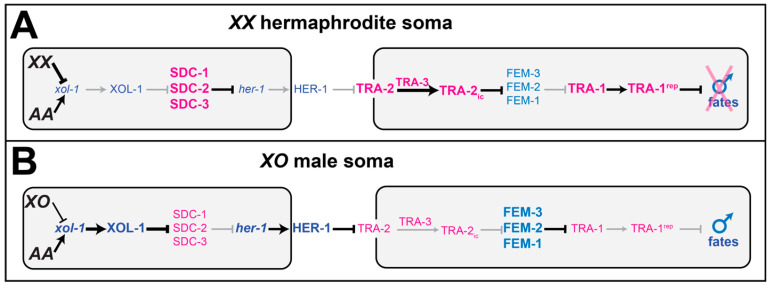
The somatic sex-determination pathway in *C. elegans. X* indicates an *X* chromosome, and *A* indicates a set of autosomes. CAPITALS indicate protein names, and *lowercase italics* indicate gene names. Items in blue promote male fates and those in red promote female ones. Black lines denote strong interactions, and gray lines denote weak ones. Arrows indicate positive regulation whereas “—|” indicates negative regulation. For details, see text. The left box denotes a signaling cell, and the right box a receiving cell. (It is possible that cells also signal to themselves.) (**A**) In *XX* animals, *xol-1* is repressed, no HER-1 is made, and TRA-2_ic_ inhibits the FEM complex. Thus, TRA-1^rep^ is free to repress male fates. (**B**) In *XO* animals, the single dose of the *X* allows XOL-1 production, which leads to HER-1 production and the inhibition of TRA-2. The absence of TRA-2_ic_ allows the FEMs to target TRA-1, leading to the expression of genes needed for male fates.

Thus, in the soma, the *X:Autosome* ratio acts through *xol-1* to control the secreted protein HER-1, which is only found in *XO* animals. HER-1 inactivates the TRA-2 receptor, which allows the FEM complex to ubiquitinate TRA-1, leading to its destruction. Finally, the absence of the TRA-1 repressor allows male genes to be expressed, so that the *XO* animals become male (Figure 3).

## 5. *C. elegans* Germline Sex Determination

All *Caenorhabditis* hermaphrodites are *XX* animals that make sperm in an otherwise female body, and switch to producing oocytes during adulthood. This pattern allows them to store sperm that can later fertilize their own oocytes. To understand how these *XX* animals make sperm requires careful analysis of how sex determination works in their germ cells. Furthermore, comparison between sex determination in this tissue and in the soma highlights many of the regulatory changes needed for self-fertility.

**Figure 4:**
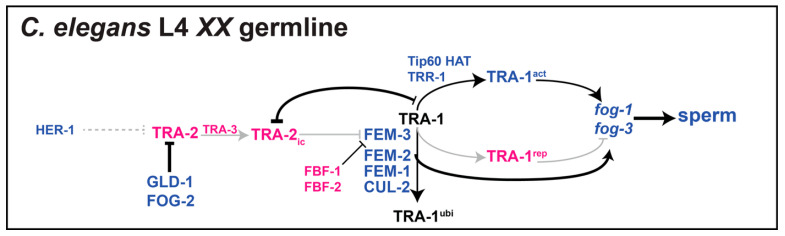
Sex determination in the L4 germ line of *C. elegans* hermaphrodites. During this period germ cells are undergoing spermatogenesis, which allows for self-fertility. Conventions are as described in Figure 3. The pathway is only shown from HER-1 downstream, since this is the critical portion for germ cell fates. Note that the core pathway is similar to that in the soma, but additional branches (TRA-2ic interacts with TRA-1 as well as FEM-3, TRA-1 makes an activator that competes with the repressor, and the FEM complex controls a second target downstream of TRA-1) and additional regulators (such as GLD-1, FOG-2, FBF-1 and FBF-2) are also critical. For details, see text.

The germline, like other tissues, is strongly influenced by the HER-1 signal and its control of the TRA-2 receptor [58]. However, this is not the whole story, since *XX* hermaphrodites make both male sperm and female oocytes. This complex goal is accomplished through several modifications to the core pathway: (1) TRA-2 and FEM-3 levels are tightly balanced in germ cells, (2) TRA-2 is subject to tissue-specific regulation in the germ line, (3) FEM-3 activity is also subject to tissue-specific regulation, (4) in addition to the cleaved repressor, TRA-1 makes a full-length activator that promotes spermatogenesis, (5) the FEM complex not only targets TRA-1, but also acts downstream of TRA-1 to promote spermatogenesis, and (6) these factors converge on two genes needed for the sperm fate, *fog-1*, and *fog-3* (Figure 4).

### 5.1. The Balance Between TRA-2 and FEM-3 in Germ Cells

Mutations that increase TRA-2 activity in germ cells cause *XX* animals to make only oocytes; these include *tra-2(mx)* mutations that alter the protein, and *tra-2*(gf) mutations that alter a regulatory site in the 3′-UTR [84,85]. By contrast, mutations that increase FEM-3 activity in germ cells cause *XX* animals to make only sperm [86]. These gf mutations alter a regulatory site in the *fem-3* 3′-UTR [87]. The tissue specificity of all these mutations suggests that germ cells are particularly sensitive to the relative levels of TRA-2 and FEM-3 activity. This model is supported by the fact that mutations that increase TRA-2 activity are suppressed by ones that increase FEM-3, and vice versa [86].

### 5.2. TRA-2 Activity Is Regulated by FOG-2 and GLD-1 in the XX Germ Line

How is TRA-2 controlled so that hermaphrodites make sperm in a female body? The *fog-2* gene is only needed for hermaphrodite spermatogenesis, so *fog-2* mutants form male/female strains of *C. elegans* [88]. Furthermore, *fog-2* is species-specific and evolved its novel function through a combination of gene duplication and divergence in the *C. elegans* lineage [89]. Thus, it is likely that *fog-2* has played a major role in the evolution of self-fertile hermaphrodites in this species.

FOG-2 is an F-box protein; to control *tra*-*2* it needs to bind to and work with the translational regulator GLD-1 [90,91]. Whereas FOG-2 has only one function, GLD-1 binds to and represses many messenger RNAs [92] including sequences in the 3′-UTR of *tra-2* [93]. The precise mechanism by which FOG-2 works with GLD-1 to regulate TRA-2 activity in *C. elegans* has not yet been discovered [94].

### 5.3. FEM-3 Activity Is Regulated by FBF-1 and FBF-2 in the XX Germ Line

The 3′-UTR of *fem-3* is also targeted by translational regulators, which are encoded by the *fbf-1* and *fbf-2* genes [95]. These are members of the diverse PUF family of RNA-binding proteins, which regulate mRNA activities [96]. The two FBF proteins regulate the translation of mRNAs involved in several different aspects of germ cell fate (reviewed by [97]). Like GLD-1, they provide an example of how the sexual fate of germ cells is in part determined by the impact of germline-specific regulators on the core sex-determination pathway. Several other translational regulators also impinge on the germline sex-determination pathway, including *nos-3*, *puf-8* and the *mog* genes; for a summary, see Ellis [98].

### 5.4. Promotion of Spermatogenesis by TRA-1 Activator and Its Co-Factors

Although TRA-1^rep^ represses genes needed for the male sperm fate [99], it is not the only isoform that acts in the germ line. In addition, full-length TRA-1 promotes spermatogenesis, competing with the cleaved form to determine whether germ cells differentiate into spermatids or oocytes [100]. In this respect, it behaves like mammalian and insect Gli proteins, which also make full-length activators and cleaved repressors. The activator appears to control only germ cell fates, since mutations that alter it do not affect development in the soma [100]. Finally, TRA-1 activator requires TRR-1, an ortholog of the TRRAP adaptor protein, and the Tip60 Histone Acetyl Transferase (HAT) complex [101].

### 5.5. Downstream Control of Spermatogenesis by the FEM Complex

Although the FEM complex regulates TRA-1 activity in the germ line as in the soma, it is also required downstream of TRA-1 to promote spermatogenesis in *C. elegans* [68,71,99]. The downstream target of this complex is currently unknown, but it is likely to be involved in the regulation of *fog-1* and *fog-3*.

### 5.6. Direct Specification of Spermatogenesis by FOG-1 and FOG-3

In *C. elegans*, two genes are specifically required for spermatogenesis in both sexes, *fog-1* [102] and *fog-3* [103]. Both are direct targets of TRA-1 transcriptional regulation [99,104]. FOG-1 is a member of the Cytoplasmic Polyadenylation Element Binding (CPEB) family of translational regulators [104,105] and FOG-3 is a Tob protein [106]. These genes appear to act in a complex to prevent the translation of large numbers of oogenic mRNAs, thus allowing germ cells to become sperm [107]. Both are conserved in other members of the genus, and are likely to form a universal part of the program that causes germ cells to initiate male spermatogenesis [47,104].

Comparing sex determination in the *C. elegans* soma and germ line suggests that some features might have been incorporated into the regulatory pathway for germ cells in order to allow hermaphrodite spermatogenesis. The most obvious example is *fog-2*, a species-specific gene needed only for hermaphrodite spermatogenesis, but additional factors might include a change in *gld-1* activity so that it could work with *fog-2*, and changes in *tra-2* and *fem-3* activities, to allow for rapid regulatory changes.

## 6. The Co-Option of Spermatogenesis in Other *Caenorhabditis* Hermaphrodites

To test this model about the specification of spermatogenesis in hermaphrodites and extend it to all three androdiecious species, it was crucial to know how sex determination works in related male/female species. For example, comparing somatic and germline regulation in *C. elegans* suggests that there is a core sex-determination pathway that is modified in the germ line to allow the production of sperm and oocytes. Is this hypothesis correct? Genetic analyses in male/female species of *Caenorhabditis* are challenging, because mutations that cause sterility in either *XX* or *XO* animals are hard to maintain in species that lack selfing. While interpreting these analyses, we also need to remember that current male/female species might also have diverged from the ancestral state, although they have been under less selective pressure to do so.

### 6.1. Determining the Ancestral Sex-Determination Pathway Using C. nigoni and C. remanei

The dioecious species *C. nigoni* is so closely related to *C. briggsae* that the two can mate and some of the hybrids are fertile [33,108]. Thus, it makes an excellent choice for studying male/female sex determination. Fortunately, gene editing using balancer mutations makes genetic analysis possible [109,110].

In *C. nigoni*, *her-1* transcript levels are high in *XO* animals but very low in the *XX* [111]. Thus, the HER-1 signal appears to work much as it does in *C. elegans*. Null mutations have been made in *Cni-tra-2*, *Cni-fem-3*, and *Cni-tra-1* to allow genetic analysis of critical genes that act downstream of HER-1 in the *C. elegans* pathway [111]. Several important results help shape our understanding of self-fertility.

**Figure 5:**
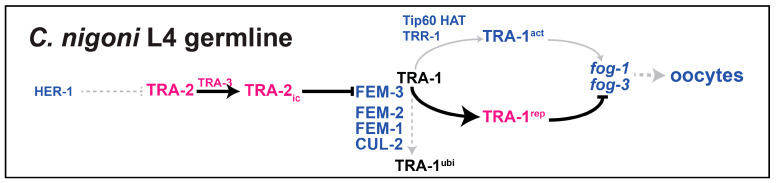
Sex determination in the L4 germ line of *C. nigoni XX* females. In these *XX* animals, all germ cells undergo oogenesis. Conventions are as described in Figure 3. The pathway is only shown from HER-1 downstream, since this is the critical portion for the hermaphrodite germ line. Note that the pathway for the germ line strongly resembles the simple linear pathway for the soma. For details, see text.

First, in *C. nigoni*, the regulatory hierarchy is very simple—*fem-3* mutations are epistatic to *tra-2* mutations in both the soma and the germ line, and *tra-1* mutations are epistatic to *fem-3* mutations [111]. Thus, the *C. nigoni* regulatory pathway is simpler than in *C. elegans*—the TRA-2/FEM-3 branch predominates in all tissues and there is no downstream requirement for the FEM complex in spermatogenesis, as seen in *C. elegans* (Figure 5). Second, mutations in *cni-tra-2* have no semi-dominant effects, unlike those in *C. elegans*. Furthermore, mutations in the *cni-fem-3* 3′-UTR regulatory site do not alter germ cells’ fates [111]. These results suggest that in *C. nigoni XX* animals, TRA-2 activity is much higher than FEM-3 activity, and vice versa in the *XO*. As a result, the *C. nigoni* pathway is more robust and less sensitive to perturbations. This conclusion is supported by the observation that *C. nigoni tra-1 XO* males make only sperm, whereas in *C. elegans*, the *tra-1 XO* mutants often switch from spermatogenesis to oogenesis during adulthood [77,112].

In the soma, *C. nigoni tra-2* mutants have imperfect male tails, just like their *C. elegans* counterparts. The *mx* domain that mediates interaction with TRA-1 is also conserved, and the two proteins are able to interact, although the effects of this interaction are unclear [111]. Finally, *C. nigoni tra-1 XX* animals make very stunted gonads and are infertile [111]. Although this differs from *C. elegans tra-1 XX* mutants, which form fertile males, careful analysis reveals minor problems even in *cel-tra-1* male gonads [113].

So far, less work has been done with *C. remanei*. The structure of its TRA-2 receptor is similar to that of *C. elegans*, the *mx* region of TRA-2 that mediates interaction with TRA-1 is conserved, and the *cre-tra-2* 3′-UTR is subject to translational regulation [114]. Furthermore, RNA interference indicates that *Cre*-TRA-2 promotes female fates [114], that *Cre*-FEM-3 promotes male gonad and tail development [115] and that *Cre*-FEM-2 promotes male gonad development [116]. Although RNA interference is not effective at eliminating all gene activities, these results suggest that in *C. remanei*, as in *C. nigoni*, the core sex-determination pathway regulates both somatic and germline fates.

The ultimate target of the pathway is also conserved in *C. remanei* germ cells. The *fog-3* promoter contains several TRA-1 binding sites, just like in *C. elegans* [47]. Furthermore, RNA interference shows that that *Cre*-FOG-3 is needed for spermatogenesis, and *Cre*-FOG-3 can rescue the *cel-fog-3* mutant phenotype in transgenic animals [47].

Finally, analysis of *C. remanei* revealed co-evolution involving TRA-2_ic_ and FEM-3, whose physical interaction is critical for *Caenorhabditis* sex determination [115]. Although the existence of this interaction within each species is conserved, the individual proteins from *C. elegans*, *C. remanei* or *C. briggsae* have diverged so much that interspecies interactions have not been detected [115]. Even exclusively within *C. remanei* populations, FEM-3 sequences are diverging rapidly [117].

### 6.2. Sex Determination in C. briggsae Reveals a Different Path to Self-Fertility

Since *C. elegans* appears to have altered the core sex-determination pathway to produce hermaphrodites, did it adopt a unique solution to this problem, or did *C. briggsae* and *C. tropicalis* evolve self-fertility through a similar set of changes?

*C. briggsae* has long been the subject of genetic investigation [46], and we know a great deal about sex determination in both its soma and germ line (reviewed by [118]). Work on XOL-1 and the SDC genes is still proceeding, but the initial determination of sex occurs along similar lines to that in *C. elegans* [53,55]. Furthermore, RNA interference shows that the HER-1 signal promotes male development in both the *C. briggsae* soma and germline, as it also does in *C. elegans* [119].

**Figure 6:**
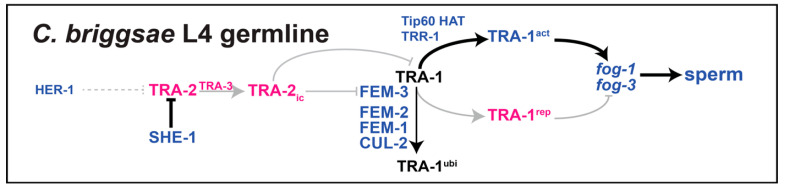
Sex determination in the L4 germ line of *C. briggsae XX* hermaphrodites. During this period germ cells are undergoing spermatogenesis, allowing for self-fertility. Conventions are as described in Figure 3. The pathway is only shown from HER-1 downstream, since this is the critical portion for the hermaphrodite germ line. Note that the pathway for the germ line depends on the TRA-1 activator branch and the FEM proteins play a little role. For details, see text.

However, within receiving cells, major differences exist in both the core sex-determination pathway and in its modifiers. The first surprising discovery was that although *fem*-2 and *fem*-3 are conserved [115,120], mutations in *C. briggsae fem-2* or *fem-3* do not produce females but instead make *XX* and *XO* hermaphrodites [48]. This result shows these genes share somatic functions with their orthologs in *C. elegans.* However, it also shows that the control of germ cell fates does not depend on the TRA-2/FEM-3 branch of the pathway (Figure 6), since *fem-2* and *fem-3* null mutants still develop as self-fertile hermaphrodites. Finally, it demonstrates that these *fem* genes do not play an essential downstream role in spermatogenesis either. However, three results suggest that the *fem* genes still play a minor role in controlling germ cell fates—the *XO* mutants make oocytes as well as sperm, as do the *tra-2*; *fem-3 XX* double mutants [48] and *fem-3* mutations increase the chance that *tra-1 XX* males will eventually make some oocytes [121].

If the TRA-2/FEM-3 branch of the pathway does not normally determine which cells make sperm and which make oocytes, how is this decision made? *C. briggsae tra-2* mutants resemble their *C. elegans* orthologs, in that *XX* animals are transformed into males that produce abundant sperm but have defective tails, so *Cbr*-TRA-2 normally prevents male germ cell fates [122]. Furthermore, *C. briggsae tra-1* mutants also resemble their *C. elegans* orthologs, in that both cause *XX* animals to become fertile males that make sperm and sometimes oocytes as well [122]. Finally, the direct interaction between TRA-2_ic_ and TRA-1 is conserved [67]. Extensive genetic evidence suggests that this interaction plays a central role in regulating spermatogenesis in *C. briggsae* hermaphrodites (Figure 6, [123]). In these nematodes, the TRA-1 activator [100], along with TRR-1 and the Tip60 HAT complex [101] is needed for spermatogenesis. TRA-2_ic_ appears to bind TRA-1 in order to prevent TRA-1 activator from promoting spermatogenesis. For example, mutations in either *cbr-tra-2* or *cbr-tra-1* that inhibit their interaction cause extra sperm production in the hermaphrodites. Thus, *C. briggsae* has increased the role of the TRA-2_ic_/TRA-1 branch of the pathway for directing hermaphrodite reproduction, whereas it has decreased the role of the FEM complex (Figure 6). By contrast, *C. elegans* germ cell fates depend to a much larger extent on the TRA-2/FEM-3 interaction and that branch of the pathway (Figure 4).

TRA-2 activity is itself regulated by SHE-1, a species-specific F-box protein that promotes hermaphrodite development in *XX* animals [124]. SHE-1 interacts with SKR-1, a part of the SCF ubiquitin ligase complex, but not with *Cbr*-GLD-1 [124]. Furthermore, *C. briggsae gld-1* mutant hermaphrodites make extra sperm [125], instead of no sperm as in *C. elegans* [90,126]. These differences are consistent with the observation that *C. elegans* FOG-2 and *C. briggsae* SHE-1 evolved from separate branches of the large F-box family and function differently. Finally, *fog-2* mutants show a clean transformation into a male/female population. By contrast, even null alleles of *she-1* are temperature sensitive, suggesting additional factors aid in in promoting hermaphrodite spermatogenesis in *C. briggsae*.

Finally, germline regulators have been recruited to the sex-determination pathway in germ cells, much like *fbf-1*, *fbf-2* and others in *C. elegans* (reviewed by [98,127]). This process not only involves differential uses of GLD-1 and its target *puf-8* (reviewed by [128]), but the control of *cbr-gld-1* by the translational regulators PUF-2 and PUF-1.2 [129].

### 6.3. C. tropicalis Also Charted a Distinct Path to Self-Fertile Hermaphroditism

Much like in *C. elegans*, the *C. tropicalis her-1 XX* and *XO* mutants develop as hermaphrodites, showing that HER-1 acts upstream of the decision to produce sperm in the *XX* germline [130]. In addition, the region of TRA-2 that binds HER-1 is conserved in *C. tropicalis*, suggesting that *Ctr*-TRA-2 functions as a transmembrane receptor for HER-1, as *Cel*-TRA-2 does in that species.

Genetic analysis reveals that *C. tropicalis tra-2 XX* and *XO* mutants develop male bodies and produce abundant sperm [130]. Although this result shows that the role of *tra-2* in germ cells is conserved, there is a surprising difference in the soma. *C. tropicalis tra-2 XX* mutants develop anatomically normal male tails, whereas *C. elegans*, *C. briggsae*, and *C. nigoni tra-2 XX* mutants develop deformed male tails that are usually missing several rays [62,111,122]. Despite their normal appearance, these *ctr-tra-2 XX* males do not attempt to mate with hermaphrodites.

**Figure 7:**
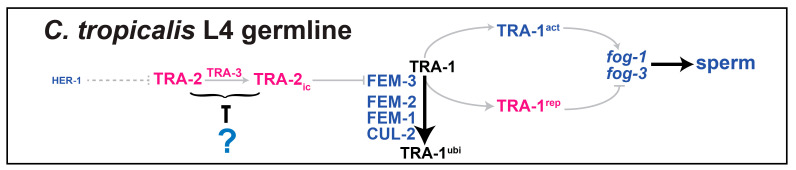
Sex determination in the L4 germ line of *C. tropicalis XX* hermaphrodites. During this period germ cells are undergoing spermatogenesis, allowing for self-fertility. Conventions are as described in Figure 3. The “**?**” identifies a regulator like FOG-2 or SHE-1 that has not yet been identified. The pathway is only shown from HER-1 downstream, since this is the critical portion for the hermaphrodite germ line. Note that germ cell fates are determined by the core pathway, as seen in somatic development or the *C. nigoni* germ line. For details, see text.

By contrast, mutations in *C. tropicalis fem-1* [130], *fem-2* and *fem-3* (Bobinski et al., manuscript in preparation) transform both *XX* and *XO* animals into true females, much as orthologous mutations do in *C. elegans*, but unlike those in *C. briggsae.* Thus, the FEM complex is not only conserved but promotes male sexual fates in both the soma and germ line. Furthermore, the fact that *C. tropicalis fem* mutants do not make sperm implies that the key regulatory interactions needed for hermaphrodite spermatogenesis occur upstream of the FEM complex. Since this decision is made downstream of the HER-1 signal, it must act either directly or indirectly on TRA-2 itself (Figure 7). This is the same point in the pathway that FOG-2 acts in *C. elegans* and SHE-1 acts in *C. briggsae*.

Finally, mutations in *C. tropicalis tra-1* cause *XX* animals to develop as males [130]. Surprisingly, these *XX* mutants do not attempt to mate with hermaphrodites, whereas the *tra-1 XO* males mate successfully. These results suggest that male mating behavior, while still controlled by the *X:Autosome* ratio, is not regulated through the core sex-determination pathway.

When studied individually, each *C. tropicalis* mutant behaves much like the orthologous sex-determination mutant in *C. elegans.* However, analysis of double mutants reveals critical differences between these species. In *C. tropicalis*, mutations in *tra-2* are epistatic to those in *her-1*, mutations in *fem-1* are epistatic to those in *tra-2*, and mutations in *tra-1* are epistatic to those in *fem-1* [130]. Mutations in *tra-1* are also epistatic to those in *fem-2* or *fem-3* (Bobinski et al., manuscript in preparation). Therefore, the FEM complex is not required for spermatogenesis in *C. tropicalis*, as it is in *C. elegans.* Instead, it must exert control largely through regulating TRA-1 activity. As a result, the *C. tropicalis* germline pathway is essentially linear (Figure 7), as in the male/female species *C. nigoni* (Figure 5), rather than highly branched, as it is in *C. elegans* (Figure 4) and *C. briggsae* (Figure 6). This result shows that complex branching is not a prerequisite for the evolution of self-fertility.

## 7. Sperm Activation in *C. elegans* Is Controlled by Redundant Signaling Programs

In addition to the adoption of male spermatogenesis by *XX* animals, the origin of self-fertile hermaphrodites required co-opting the ability to activate spermatids, so that they can locate and fertilize oocytes. In males, inactive spermatids only become motile spermatozoa during ejaculation, after which they migrate from the uterus to the spermathecae of their *XX* partners, and eventually fertilize passing oocytes (reviewed by [131,132]). By contrast, hermaphrodites make spermatids when young and store them for later use in fertilizing oocytes (reviewed by [44]). Since inactive spermatids cannot seek out oocytes or maintain their positions in the spermathecae, hermaphrodite spermatids must become motile without exposure to male ejaculate. Instead, activation in hermaphrodites occurs when their spermatids are pushed into the spermatheca by maturing oocytes, where they receive an activating signal (reviewed by [131,132]). How does this signal work?

**Figure 8:**
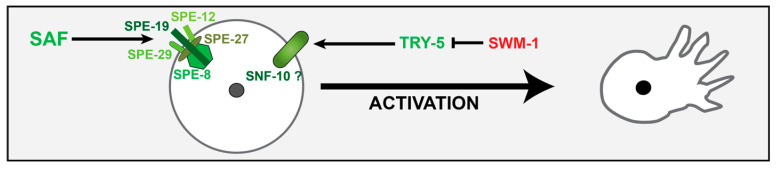
*C. elegans* sperm are activated by redundant signals in males. In *C. elegans*, round spermatids must be activated so that they extend pseudopods and crawl towards oocytes. An unknown Sperm-Activating Factor (SAF) acts through SPE-8 and associated proteins at the plasma membrane of the spermatid to initiate spermiogenesis. Extracellular TRY-5 protease acts independently, by cleaving SNF-10 or regulating its activity. SWM-1 blocks premature activation by TRY-5.

Genetic analysis of *C. elegans* has identified a group of genes needed specifically for hermaphrodite sperm activation (reviewed by [41]). First, mutations in *spe-8* [133], *spe-12* [134], *spe-19* [135], *spe-27* [136], *spe-29* [134] and *spe-43* [137] prevent hermaphrodite self-fertility. The problem caused by each of these mutations is that hermaphrodite spermatids do not activate and are soon lost, preventing self-fertilization. By contrast, the male spermatids activate and function normally. The problem with hermaphrodite activation must involve signaling, since male seminal fluid can *trans*activate mutant hermaphrodite sperm during mating, restoring their ability to fertilize oocytes. Thus, these six genes define a hermaphrodite sperm-activation pathway (Figure 8, left).

Each gene in this *spe-8* group makes a protein found at or near the sperm membrane. Hence, these genes seem likely to mediate the response of spermatids to an external activation signal. However, we still do not know what the Sperm-Activating Factor is, although it seems likely to be expressed by spermathecal cells in hermaphrodites.

Surprisingly, *C. elegans* has a second sperm activation signal, TRY-5, which was first identified in males [138,139]. Prior to mating, TRY-5 in the male gonad is inhibited by SWM-1 [140], but during ejaculation it is secreted into the seminal fluid, where it activates spermatids (Figure 8, right). Its target might be the membrane protein SNF-10 [141]. Although *try-5* males are fertile, *spe-8; try-5* double mutants are sterile [138], so the two sperm activation pathways are redundant in males. Genetic analyses show that both pathways are used in *C. briggsae* males for sperm activation as well, so they must have been present in their common ancestor with *C. elegans* [39].

## 8. Each Hermaphroditic Species Co-Opted One of Two Sperm Activation Pathways for XX Animals

Since the ancestral *Caenorhabditis* species had two redundant sperm activation programs, how was this male trait co-opted for use in hermaphrodites? This question was resolved using gene editing to analyze each pathway in *C. briggsae* and *C. tropicalis*, the other androdiecious species.

**Figure 9:**
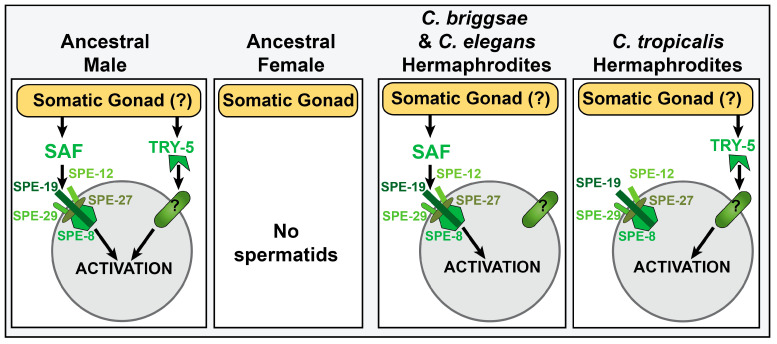
Hermaphrodite co-option of signaling pathways to activate self-sperm. (**Left**) Two redundant pathways activate sperm in *Caenorhabditis* males. No spermatids or signal are present in females, so male spermatids can only be activated by male seminal fluid. (**Right**) Hermaphrodites co-opted one or the other pathway to activate self-spermatids.

Like *C. elegans*, *C. briggsae* hermaphrodites use only the *spe-8* pathway to activate their sperm, [39]. By contrast, *C. tropicalis* hermaphrodites use only the TRY-5 pathway [39]. Thus, each androdiecious species co-opted one of the two male sperm activation pathways for use in hermaphrodites (Figure 9). This change could have occurred because a regulatory mutation allowed the *XX* spermathecae to express one of the male signals, or perhaps because a mutation caused them to stop producing an inhibitor of a signal that was already present. Finally, either activation pathway is sufficient for *XX* self-fertility, because none of these species co-opted both pathways for use in hermaphrodites.

## 9. What Is Required for Self-Fertility to Evolve in These Nematodes?

Since these three androdiecious species each evolved self-fertility independently, we are now in a position to paint a general picture of how this complex trait evolved.

### 9.1. Influence of the Environment on Self-Fertility—Selection for Colonizing Ability

**Figure 10:**
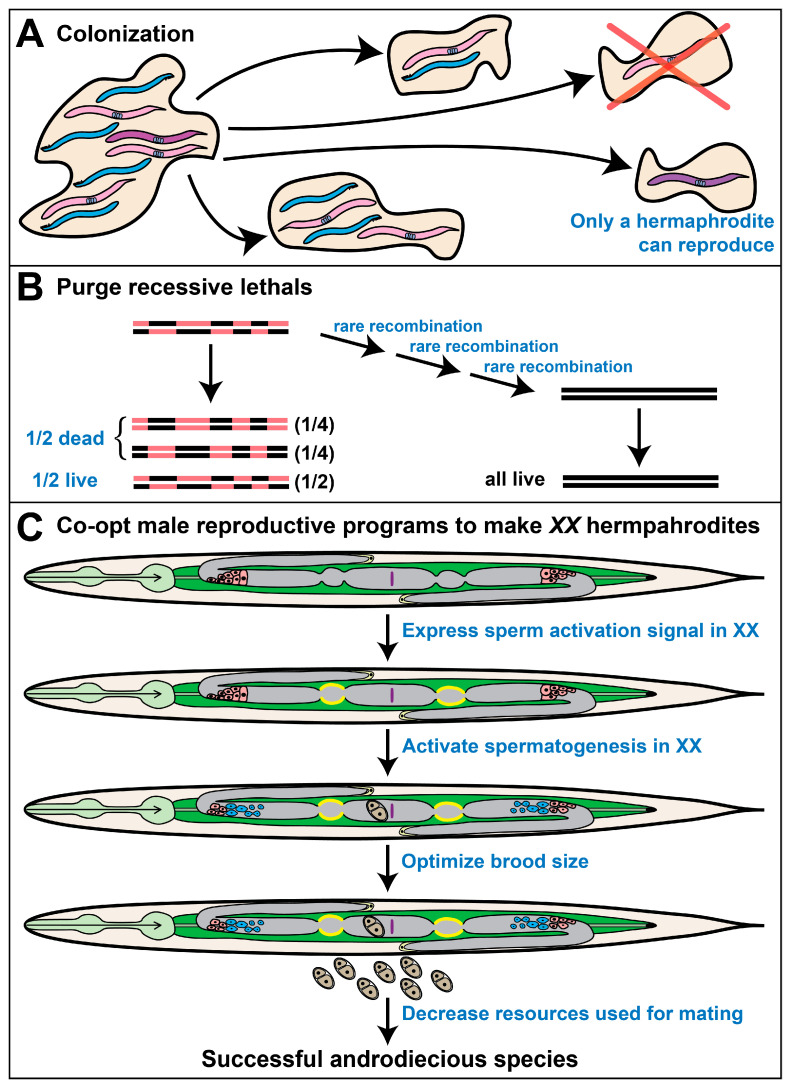
Steps involved in the origin of androdiecious mating systems in *Caenorhabditis.* (**A**) *Caenorhabditis* nematodes exploit ephemeral food sources in a boom-and-bust population cycle. If the distance between these food sources changes so that only single animals succeed in colonization, self-fertile hermaphrodites will have a strong selective advantage. Blue indicates males, pink females and purple hermaphrodites. (**B**) Diagram of a single chromosome in a heterozygous hermaphrodite. If the red regions carry recessive lethal mutations, inbreeding by selfing carries a high cost until recombination can produce chromosomes that lack these mutations. (**C**) Developmental changes in the origin of self-fertile hermaphrodites. The order of the first two steps is uncertain.

The environment shapes the selective forces that drive evolution. Because self-fertile hermaphrodites do not need a mate to reproduce, environmental conditions requiring dispersal over long distances could decrease the chance that males and females both arrive at the same location at the same time; this would favor the evolution of self-fertility (Figure 10A, [142]). For example, a study of the re-population of Northern Europe after the last ice age by different populations of the tadpole shrimp *Triops cancriformis* showed that hermaphrodites predominate in colonized areas, and male/female populations in ancient refuges [143].

Most *Caenorhabditis* nematodes grow and reproduce on small, ephemeral populations of bacteria found on rotting plants [33,144]. The boom-and-bust nature of these food sources puts a premium on rapid growth and dispersal, and the dauer larval stage is specialized for the challenge of moving to new food sources when old ones are exhausted (reviewed by [145]). Given this, the efficiency of hermaphrodites at colonization might account for the broader global distribution of the three androdiecious species [146,147]. For example, tests in the wild showed that *C. briggsae* exceled at reaching and exploiting new locations, as compared to male/female species [148]. To reach these new locations, most *Caenorhabditis* probably use phoresis, attaching to small insects and other animals for transportation, so changes in the distribution of these vectors might also affect colonization [148,149,150]. Additionally, human global migrations could have been a critical vector.

### 9.2. Influence of the Environment on Self-Fertility—Overcoming Inbreeding Depression

The need for efficient dispersal and colonization not only favors self-fertile hermaphrodites but also creates a major challenge for nematode populations. The populations of gonochoristic members of this genus like *C. brenneri* show extremely high genetic diversity, as one might expect for large male/female populations [151]. This genetic diversity usually leads to significant lethality if small populations begin inbreeding [152,153,154]. Hence, switching to a reproductive strategy that relies on maximum dispersal and self-fertility could result in small, inbred populations that rapidly die out. Analyses of wild isolates of *C. tropicalis* and *C. elegans* show that these populations have much lower genetic diversity and display outbreeding depression, suggesting that they purged many recessive lethal mutations from their gene pool [155,156]. This purging process would have been a significant challenge during the origin of androdiecious mating systems (Figure 10B). Perhaps the establishment of inbred populations of androdiecious nematodes also helped establish drive elements and segregation distorters that make outcrossing even more dangerous [157,158,159].

### 9.3. Genetic and Anatomical Preconditions for the Evolution of Self-Fertility in Caenorhabditis

Natural selection probably favors self-fertility in many colonizing groups that have not produced self-fertile hermaphrodites. By contrast, *Caenorhabditis* has produced three such species. Thus, some ancestral traits in these nematodes might simplify this evolutionary transition, and these traits might be lacking in many other animals. For example, the presence of the spermathecae in female nematodes (Figure 2) makes it simple for these species to use hermaphrodite sperm, since they are designed for sperm storage and ideally located to receive self-sperm. The ease with which this structure can be adapted for use in hermaphrodites probably made the developmental transition less complex [38].

Second, the evolution of androdiecious species required *XX* females to be replaced by *XX* hermaphrodites, whereas *XO* animals remained male. This *XX*/*XO* chromosomal sex-determination system also simplified the evolution of hermaphrodites, because there is no *Y* chromosome carrying male genes, as is seen in many groups of animals (reviewed by [160]). Hence, the *XX* individuals in ancestral male/female species possess all the genetic information needed to produce sperm as well as oocytes.

A more speculative precondition involves the structure of the regulatory pathways that need to be co-opted. Since somatic and germline sex determination are similar in many *Caenorhabditis* species and use a common signal transduction pathway, all that is needed for *XX* animals to activate male spermatogenesis is the recruitment of regulators that temporarily block female fates in the germ line. In all three androdiecious species, these new regulators appear to target TRA-2 (Figure 4, Figure 6 and Figure 7). Perhaps the fact that TRA-2 is located at a branch point and can interact directly with TRA-1 makes it an ideal target for controlling downstream sexual fates. If so, the structure of the sex-determination pathway in these nematodes makes TRA-2 a ‘hot spot’ for regulation, much like certain genes are ‘hot spots’ for mutations that cause evolutionary change (reviewed by [161]).

A further regulatory precondition is that nematode germ cells can change sexual fates without inducing cell death. Changing germ cell fates in many other species like *Drosophila*, instead results in cell death or non-functional gametes (reviewed by [162]).

### 9.4. Stepwise Co-Option of Male Traits Might Have Facilitated the Evolution of Hermaphrodites

Thus, all *Caenorhabditis* species inherited features that facilitated hermaphroditism, and some might have been subject to selective pressure favoring self-fertility, as the environment changed to separate food sources by greater distances. What genetic changes were needed to initiate hermaphrodite reproduction?

*Decreasing robustness in sex determination:* Genetic analyses of *C nigoni* suggest that it is difficult to produce *XX* animals that make both sperm and oocytes by mutations in sex-determination genes [111]. Even hybrids with its sister species *C. briggsae* almost always develop as females rather than hermaphrodites [108,111,163]. So far, the only method known to cause *C. nigoni XX* animals to make both sperm and oocytes is RNA interference against *tra-2* [38,39]. This approach might facilitate a switch to oogenesis by exhaustion of the *tra-2* double-stranded RNA during aging.

These observations highlight two requirements for the transition from *XX* females to *XX* hermaphrodites. First, the sex-determination pathway is robust in the male/female species *C. nigoni*, so it seems unlikely that a single mutation could change germ cell fates in a productive way. Second, any changes that do occur must be limited in effect, so that *XX* animals eventually make oocytes as well as sperm. A simple hypothesis is that, prior to the emergence of *XX* hermaphrodites, male/female populations acquired mutations that lowered the robustness of sex determination in the germ line. These mutations could have accumulated over a long period of developmental system drift [6], and only exerted their effects when the optimal combinations came together in isolated subpopulations. If these mutations had the effect of lowering the disparity between TRA-2 and FEM-3 activities in the germ line so that other regulatory changes could strongly affect germ cell fates, they might have solved both the problems described above. Current evidence suggests that *C. elegans* might have taken this path, and that *C. briggsae* effectively eliminated the need for TRA-2/FEM-3 interactions in germ cells. We do not yet know how this problem was solved in *C. tropicalis*.

*Co-option of sperm activation signals for use in XX animals:* Normally, the two sperm activation signals are expressed in cells of the male somatic gonad, as was observed for *C. elegans* TRY-5 [138] and is suspected for the unidentified *spe-8* signal. Thus, promoter changes that induce expression of either signal in the XX spermathecae would allow hermaphrodite sperm activation (Figure 10C).

*Co-option of the spermatogenesis program for use in XX animals*: To express male spermatogenesis genes, *XX* nematodes must alter the activity of genes that specify spermatogenesis during the development of the germ line. These genes appear to be *fog-1* and *fog-3* in all species, but also the three *fem* genes in *C. elegans.* Hence, regulatory changes that increase TRA-1 activator levels, or decrease those of TRA-1 repressor, appear to be required. In addition, *C. elegans* needed changes that increased FEM activity. In all three species this transition seems to have been accomplished by novel regulation of TRA-2 in the germ line, with each androdiecious species adopting a unique method. Finally, these changes should not be so extreme that *XX* animals are unable to begin making oocytes when they mature. So far, it is unclear what factors cause the switch from spermatogenesis to oogenesis in *XX* animals, but it is likely to be facilitated by a decrease in robustness in the regulatory pathway.

Although there were three distinct paths used to alter the sex-determination system in this genus, there are in theory far more dramatic changes that could have been made to the regulatory pathway. Many of these possibilities were tested and described by Hodgkin [164]. They include alterations to the chromosomal signal that initially determines sex, and even changes that eliminate this system altogether. These types of systems were not observed in *Caenorhabditis* but might exist in other nematodes (below). Other systems described by Hodgkin rely on heterozygosity for unusual alleles of sex-determination genes, changes in epistasis, or the possibility that temperature-sensitive alleles could be central to environmental sex determination.

Why do both identified regulators of *XX* spermatogenesis (*fog-2* and *she-1*) encode F-box proteins? The large size and diverse nature of this gene family in *Caenorhabditis* [165] might make it particularly fruitful for duplication and divergence. This would facilitate the evolution of novel proteins (reviewed by [166]).

*Optimization of brood size*: Self-fertile *C. remanei* and *C. nigoni XX* animals produced by RNA interference have very small broods [38,39]. By contrast, *C. elegans*, *C. briggsae* and *C. tropicalis* hermaphrodites produce 150–300 self-progeny. Competition experiments show that the size of the self-brood is under strong selective pressure [167]. Too few self-progeny would prevent a hermaphrodite from competing with other hermaphrodites. However, investing in too many sperm and producing an extremely large brood delays oogenesis and reproduction, which is also a losing strategy. Thus, the precise number of self-sperm would have been under selection once populations committed to an androdiecious mating system.

*Decline in resources used for mating*: Despite the persistence of males in androdiecious species like *C. elegans*, many aspects of male function and behavior have declined.

One striking example is that the evolution of self-fertility in *Caenorhabditis* has been accompanied by decreasing genome size [168]. Specifically, the genomes of the self-fertile species *C. elegans*, *C. briggsae*, and *C. tropicalis* are 20–40% smaller than those of male/female species like *C. nigoni*, *C. remanei*, *C. japonica*, *C. brenneri*, and *C. sinica* [168,169]. Although similar changes in genome size have been observed between self-fertilizing and outcrossing plant species [170], this pattern is not universal. In *Caenorhabditis*, the decrease is broad but also includes the loss of specific classes of genes. For example, when *C. briggsae* is compared with its male/female relative *C. nigoni*, sperm competitiveness genes, including the male secreted short (*mss*) gene family, are missing [169]. These *mss* genes are also found in other male/female species, like *C. japonica* and *C. afra*. By contrast, they are missing from all three hermaphroditic species. This difference is likely the result of independent gene losses following the origin of self-fertility.

Why are these sperm-competitiveness genes preserved in males from dioecious species of *Caenorhabditis*, but not in males from the androdiecious species, which still need to compete against sperm from other males or hermaphrodites? To answer this question, Yin et al. observed how the *mss* genes affected crosses involving both outcrossing and self-fertilizing nematodes [169]. They found that males from the gonochoristic species *C. remanei*, in which the *mss* genes had been deleted, made sperm that competed poorly with those from wildtype males. However, these mutant sperm fertilized oocytes normally if there were no competitors [169]. Conversely, the introduction of *mss* genes into *C. briggsae* caused sperm from the *mss*(+) males to outcompete those from wildtype males and from hermaphrodites. Thus, genes that mediate sperm competitiveness are retained in male/female species but appear to be superfluous and rapidly lost in androdiecious ones. It is unclear if this loss is caused by the random accumulation of mutations in the absence of selective pressure to maintain the *mss* genes, or is in itself favorable, as might occur in the loss of vision in cave fish (reviewed by [171,172]).

The size of male sperm also decreases in androdiecious species, when compared with sperm from gonochoristic males [27,173]. In interspecies crosses, the larger sperm from gonochoristic males physically displace smaller sperm from androdiecious males and are thus more competitive [174]. Even just within *C. elegans*, decreasing sperm size is associated with decreasing competitiveness [175,176]. Finally, there is also a difference in size and competitive ability within each androdiecious species between male sperm and hermaphrodite self-sperm [173]. Unlike the other examples, this sexual difference appears to be caused by a developmental bias, because spermatids that develop in an otherwise female gonad do not grow as robustly as those growing in males [27].

Why are some competitive male sperm traits, including size, motility and invasiveness, either missing or reduced in self-fertile *Caenorhabditis* species? The answer appears to lie in sexual conflicts. In *Caenorhabditis*, male sperm can induce sterility and shorten hermaphrodite lifespan in inter-species crosses [174]. This is primarily accomplished through the displacement of conspecific sperm, as well as by the invasion of the ovary, breaching the gonad, and infiltration of other tissues by these inter-species sperm. Hermaphrodites of androdiecious species appear more vulnerable to this sperm-mediated harm than do females, although even conspecific sperm can be damaging. Thus, self-fertile species might optimize for hermaphrodite lifespan and fertility by spending fewer resources defending against male sperm, which are rarely encountered in the wild. Additionally, their males would need to invest less in competition against other male sperm.

A decline in sexual function is not limited to males. For example, *Caenorhabditis* females are normally attracted to males, whereas hermaphrodites are not [177]. These changes affect specific chemotactic responses, primarily involved in mating. Other complex interactions between the sexes still exist in the androdiecious species, many involving pheromone signaling [178].

One trait long thought to be produced by selection, because it favors male/hermaphrodite mating, is the small size and poor competitiveness of hermaphrodite self-sperm. However, it appears this trait is instead caused by a developmental bias rooted in the inability of the female gonad to nurture the same robust growth of spermatocytes as the male gonad [27].

## 10. Comparing *Caenorhabditis* Mating Systems with Other Nematodes

To what extent can these observations about the evolution of self-fertility in *Caenorhabditis* be generalized? Intriguing work is now being done with several other groups of nematodes that provide illuminating comparisons.

### 10.1. Androdioecy in Pristionchus

Outside of *Caenorhabditis*, the leading nematode for genetic, developmental and ecological research is *Pristionchus pacificus* [179]. Like *C. elegans*, the species has five autosomes and an *X* chromosome, with the *XX* animals developing as self-fertile hermaphrodites and the *XO* as males. However, many of the similarities with *C. elegans* are convergent. Detailed analyses of *Pristionchus* suggest that the ancestral species had six autosomes and an *X* chromosome, with the *XX* animals being female and *XO* male [180,181]. Furthermore, numerous chromosomal fusions have subsequently occurred, some of which produced pseudo-*XY* sex-determination systems, in which the *X* to autosome fusion acts like an *X* chromosome, and the unfused autosome like a *Y*. Thus, *XO* mating systems are not essential for self-fertility. Surprisingly, two androdiecious species, *P. mayeri* and *P. entomophagous*, have six chromosomes including an ancestral *X*, but lack chromosomal sex determination altogether; instead, sex is specified stochastically, with males produced at a low frequency by an unknown mechanism [181].

Despite these differences in the use of chromosomal signals, the role of TRA-1 has been conserved between *Caenorhabditis* and *Pristionchus*. Mutations in *tra-1* cause all animals to develop as males in both *P. pacificus* (which uses an XO system, [182]) and *P. mayeri* (which determines sex stochastically, [181]). Although homologs of other *Caenorhabditis* sex-determination genes have been detected in *Pristionchus* species, the key genes *tra-2* and *fem-3* have not [181]. Thus, we do not know how *Pristionchus tra-1* is regulated, nor how this regulation has been modified to produce self-fertile hermaphrodites.

All of these species share the use of spermathecae as sperm storage organs with *Caenorhabditis*, as well as their position between the ovary and uterus. Thus, this anatomical feature could be a critical precondition for changes in nematode mating systems. However, results from *Pristionchus* suggest that the *XO* chromosomal sex determination found in *Caenorhabditis* is not essential for the evolution of self-fertility, since at least two species make males and hermaphrodites through stochastic means. Indeed, it is even possible that *P. pacificus* developed its *XO* system during or after the origin of self-fertility, since the phylogeny is not clear on this point [181]. Nonetheless, the hypothesis that having *XX* animals that carry all the genes needed for male reproduction facilitates the origin of self-fertility still applies. In the pseudo-*XY* species of *Pristionchus*, the “*X*” chromosome is a fusion and the “*Y*” is an ancestral autosome, so the “*XX*” animals indeed carry all genetic information for that species. Similarly, all members of the stochastic species are homozygous for all chromosomes.

### 10.2. Three Sexual Morphs in Auanema

Self-fertile hermaphrodites are not only found in androdiecious species like *C. elegans*. Members of the genus *Auanema* have a more complex mating system with three sexual morphs—*XX* females, *XX* hermaphrodites, and *XO* males [183,184]. The hermaphrodites develop through the alternative dauer larval stage and are specialized for dispersal, whereas the females are specialized for rapid population growth in optimal conditions [184,185]. These results strongly support the hypothesis that self-fertility is favored because it facilitates colonization. So far, all known species in this genus—*A. rhodensis*, *A. freiburgensis* and *A. melissensis*—share this mating system [183,186,187]. Thus, it is unlikely to reflect a brief transitionary period between male/female and male/hermaphrodite mating systems. Instead, it appears to represent a stable solution for balancing the needs for colonization in harsh conditions and for rapid growth when food is abundant.

Members of this genus illuminate two further aspects of sex determination and self-fertility. First, they undergo biased patterns of chromosomal segregation during spermatogenesis, ensuring that the favored sexes are produced by crosses or by selfing [188,189,190]. Furthermore, complementary changes to chromosome segregation during oogenesis occur in females and hermaphrodites [191]. These alterations of meiosis allow the animals to use *XX*/*XO* chromosomal sex determination, but produce unusual ratios of males, females and hermaphrodites (reviewed by [192]). However, the core *XX*/*XO* system would have facilitated the origin of *Auanema* hermaphrodites.

Second, one sex-determination mutant has already been isolated in *A. rhodensis*, the masculinizing mutant *mas-1(brz3)* [193]. This recessive mutation transforms *XX* animals into fertile males. Although *C. elegans* and *C. briggsae tra-1 XX* mutants are also fertile males, they show weak feminization in the germ line that is not observed in the *mas-1* animals. So far, the molecular nature of *mas-1* and of additional sex-determination genes remain unknown. Thus, the regulatory changes needed to produce self-fertile animals in *Auanema* also remain unknown and are likely to require mutagenesis and genetic mapping to define.

Some entomopathogenic nematodes, which parasitize insects, also feature three sexual morphs like those in *Auanema.* These include members of the genus *Heterorhabditis* [194,195,196] and at least one species of *Steinernema* [197,198].

### 10.3. Parthenogenesis

Rare nematodes have evolved asexual reproductive systems from male/female ancestors. For example, *Diploscapter pachys* lacks males and does not use sperm for female reproduction [199]. Unexpectedly, members of this genus have a single large chromosome, produced by the fusion of all ancestral chromosomes [199,200]. Despite reproducing parthenogenetically, some of these species occasionally produce males [199] and genomic analyses suggest that rare sequence introgressions have occurred that would have required mating [201]. That even parthenogenetic species might retain the ability for occasional male mating suggests that a critical factor sustaining androdiecious nematodes is the ability to exchange genetic information through crosses involving males.

### 10.4. More Distant Relatives and Parasitic Nematodes

Although most of our conclusions hold when extended to the free-living nematodes described above, more distant relatives might lack the favorable developmental constraints we describe. Furthermore, most parasitic nematodes develop in very different environments, in which conflicts with the host often predominate. These environments might select for high genetic diversity, which is not typically found in populations of self-fertile hermaphrodites.

## 11. Conclusions

The evolution of self-fertile hermaphrodites in *Caenorhabditis* involved a long sequence of events that illuminates all aspects of evolutionary theory. Strong selective pressure favoring efficient colonization drove the process, probably in response to environmental changes that decreased the availability of rich bacterial food sources. Small subpopulations might have undergone a lengthy process in which recessive lethal and sterile mutations were purged to eliminate inbreeding depression. This change could have occurred by necessity after hermaphrodites arose and began selfing or in the smaller and smaller male/female subpopulations produced by environmental changes that favored colonization. Because *Caenorhabditis* nematodes have spermathecae located in a favorable position to store self-sperm, and because they use an *XX*/*XO* mating system, regulatory changes that favor hermaphrodite spermatogenesis and sperm activation could have rapidly produced self-fertile animals. These changes probably involved mutations that decreased the robust nature of sex determination in germ cells, mutations that altered TRA-2 activity so that *XX* animals could produce sperm as well as oocytes, and mutations that caused the expression of a sperm activation signal in the spermathecae. The precise order of these three steps is unknown, but we favor models in which mutations that lowered robustness and caused *XX* sperm-activation signals occurred first. These two developmental changes would have few negative consequences and would provide a favorable situation for further mutations that led to male spermatogenesis in *XX* animals [38]. Finally, newly evolved hermaphrodites would be under strong selective pressure to optimize their brood size by further altering the sex-determination pathway, and under reduced pressure to maintain genes primarily involved in mating.

With this rich background available, we are now poised to identify the molecular changes in promoters and proteins that caused these developmental transformations. Regulatory sites and broad models can be tested with gene-swap experiments using related species. Finally, experimental evolution is a promising new area of nematode genetics and could test many of the hypotheses presented here (reviewed by [202,203]). With our current ability to compare multiple species, these approaches could help us move from pathway-centered models to a precise understanding of the changes needed to convert ancestral females into hermaphrodites.

## Data Availability

Data sharing is not applicable. No new data were created or analyzed in the preparation of this review article.
